# Daily Chronic Intermittent Hypobaric Hypoxia Does Not Induce Chronic Increase in Pulmonary Arterial Pressure Assessed by Echocardiography

**DOI:** 10.1155/2018/9649716

**Published:** 2018-04-01

**Authors:** Jeremias Götschke, Pontus Mertsch, Nikolaus Kneidinger, Diego Kauffmann-Guerrero, Jürgen Behr, Rudolf Maria Huber, Frank Reichenberger, Katrin Milger

**Affiliations:** ^1^Department of Internal Medicine V, Ludwig-Maximilians-University of Munich, Munich, Germany; ^2^Comprehensive Pneumology Center (CPC-M), Member of the German Center for Lung Research (DZL), Munich, Germany; ^3^Asklepios Fachkliniken München Gauting, Gauting, Germany

## Abstract

Chronic hypoxia causes pulmonary vascular remodeling resulting in persistently increased pulmonary arterial pressures (PAP) even after return to normoxia. Recently, interest in chronic intermittent hypobaric hypoxia (CIHH) was raised because it occurs in subjects working at high altitude (HA) but living in lowland. However, effects of daily CIHH on PAP are unknown. In this pilot study, we included 8 healthy subjects working at (2650 m) each workday for 8-9 h while living and sleeping at LA and 8 matched control subjects living and working at LA. Cardiorespiratory measurements including echocardiography at rest and during exercise were performed at LA (Munich, 530 m) and HA (Zugspitze, 2650 m). Hemoglobin was higher in CIHH subjects. LA echocardiography showed normal right and left cardiac dimensions and function in all subjects. Systolic PAP (sPAP) and tricuspid annular plane systolic excursion (TAPSE) at rest were similar in both groups. Resting blood gas analysis (BGA) at HA revealed decreased pCO_2_ in CIHH compared to controls (HA: 28.4 versus 31.7 mmHg, *p*=0.01). During exercise, sPAP was lower in CIHH subjects compared to controls (LA: 28.7 versus 35.3 mmHg, *p*=0.02; HA: 26.3 versus 33.6 mmHg, *p*=0.04) and peripheral oxygen saturation (SpO_2_) was higher. In sum, subjects exposed to CIHH showed no signs of pulmonary vascular remodeling.

## 1. Introduction

Acute and chronic physiologic reactions to altitude are observed starting at altitudes of 1400 m and have been studied in detail in the last decades [1]. Maladaptive diseases such as acute and chronic mountain sickness or high-altitude pulmonary edema and hypertension are observed at altitudes above 2500 m (high altitude) [2, 3]. The main causative factor for adaptive and maladaptive reactions is hypoxia. However, atmospheric pressure is also relevant, as differences between reactions to normobaric and hypobaric hypoxia have been demonstrated [4–7].

Acute hypoxia causes hypoxic pulmonary vasoconstriction (HPV) leading to an increase in pulmonary arterial pressure (PAP) with the level of altitude being inversely related to arterial oxygen saturation (SaO_2_) and directly related to PAP [8]. Mean (m)PAP increases from 10 to 15 mmHg at lowland to values of 20–25 mmHg at altitudes of 2600–3600 m [9, 10]. Under chronic hypoxia, vascular remodeling occurs in the pulmonary arteries with thickening of the adventitia and media and muscularization of formerly nonmuscularized precapillary vessels, causing a sustained increase in PAP even weeks after return to normoxia [11].

PAP increases upon exertion because of rising cardiac output (CO). Yet, this increase is moderate in healthy subjects even under maximal exercise, because vasodilation and vascular recruitment are decreasing pulmonary vascular resistance (PVR) at the same time. A disproportionately high rise in PAP under exertion is considered a sign of early stages of pulmonary vascular remodeling and disease [12]. At altitude, PAP rises more sharply with the increase in CO upon exercise compared to sea level [13]. Thus, exercise at high altitude may unmask pulmonary vascular alterations that might not be noted at rest or at lowland.

A further important physiologic response to acute hypoxia is a rise in breathing rate and tidal volume, termed hypoxic ventilatory response (HVR). This hyperventilation increases alveolar and arterial oxygen pressure and decreases carbon dioxide pressure and is sustained during chronic exposure in healthy high-altitude dwellers, while a decrease in hyperventilation during chronic hypoxia is seen as an initial mechanism of maladaptation and development of chronic mountain sickness (CMS) [8].

In the course of the touristic exploitation of the Alps and other mountains as well as other activities such as research stations, an increasing number of people are working in higher altitudes while living in lowland and commuting between these altitudes every workday by gondola lift or cable car. Recently, interest in periodic exposure to high altitudes, also known as chronic intermittent hypobaric hypoxia (CIHH), as occurring in various working conditions, was raised. It has been shown that adjustments of ventilatory and cardiovascular responses, as well as hemoglobin (Hb) levels are similar to those observed under chronic hypoxia, but the acclimatization may take years instead of months [14, 15]. Interestingly, increased PAP at sea level with high prevalence of borderline pulmonary hypertension (PH) or, to a lower degree, even manifest PH was found in subjects exposed to CIHH for over 12 years [16].

So far, studies investigating CIHH focused on exposure–nonexposure durations of one to several weeks as occurring in mining or military workers [14, 16, 17]. Yet, the effect of daily CIHH is unknown. Therefore, we studied the effect of daily CIHH on cardiopulmonary physiology in subjects commuting every workday from 700 m to 2656 m asl for several years, focusing on the question whether this exposure induces pulmonary vascular remodeling and chronic increase in pulmonary arterial pressure. We further performed measurements during exercise to increase sensitivity of detecting pulmonary vascular alterations.

## 2. Methods

### 2.1. Study Population

In the CIHH group, we included 8 healthy Caucasian subjects (4 men and 4 women) working at high altitude (2650 m) on the Zugspitze (Wetterstein Mountains, Germany) with daily ascension by gondola lift or cable car from 700 m and an average high-altitude stay of 8-9 h per day, 5 days a week. Work activities of the staff were administrative work (*n*=2), engineer and technician (*n*=3), waitress and sale assistant (*n*=3). Controls were matched according to age, gender, BMI, health, and physical activity status. All controls were living and working in lowland (Munich, Germany, 530 m asl) without any previous chronic or chronic intermittent exposure to high altitude and no acute hypoxia within the last 3 months. All subjects had previously traveled to similar heights and reported no history of acute mountain sickness. Work activities of controls were technician (*n*=3), nurse (*n*=2), physician (*n*=1), housewife (*n*=1), and secretary (*n*=1). Prior written informed consent was obtained from all subjects. The study was approved by the institutional review board (IRB) of the University of Munich (number 328-16).

### 2.2. Study Design

**Figure 1:**
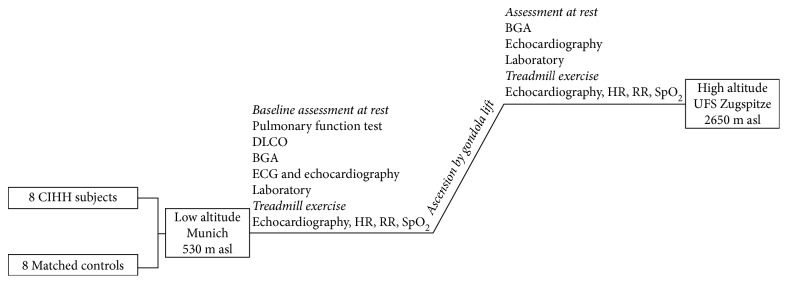
Study overview.

The study overview is presented in Figure 1. Baseline low-altitude investigations of both groups were performed in Munich (low altitude 530 m asl; Department of Internal Medicine, University Hospital of the Ludwig-Maximilians-University, Munich, Germany). High-altitude measurements were performed two weeks later at the air-conditioned Environmental Research Station Schneefernerhaus (UFS, Zugspitze, Germany) at an altitude of 2650 m, located 300 m below the top of Germany's highest mountain, Zugspitze. The station was effortlessly reached by all participants by gondola lift and measurements started 2 h or later after arrival at high altitude. Blood withdrawal at low and high altitude was performed shortly before the exercise test.

### 2.3. Measurements

All measurements at low and high altitude were carried out at room temperature (19–22°C).

Baseline measurements at low altitude included venous blood analysis for hemoglobin, ferritin, pro-BNP, endothelin-1, pulmonary function testing using bodyplethysmography, diffusion capacity for carbon monoxide, cycle ergometer exercise with capillary blood gas analysis, and echocardiography. Plethysmography was performed using Jaeger MasterScreen Body/Diff (Carefusion SN696373; Hoechberg, Germany). Peripheral oxygen saturation (SpO_2_) was measured at fingertip (Criticare 504-US). Blood gas analysis (BGA) was performed from the arterialized earlobe sample using the Rapidpoint 405 (Siemens Healthineers, Erlangen, Germany). Ergometry was performed as bicycle ergometry in semi supine position tilted toward the left side for better visualization of the cardiac structures as described elsewhere [18]. We used a step-protocol using predefined steps of 50 W every two minutes at low altitude and 25 W every two minutes at high altitude. In addition to echocardiography, subjects were monitored during exercise by continuous measurement of heart rate and SpO_2_ as well as blood pressure measurement every 2 min. All echocardiography measurements were performed by the same, experienced investigator (FR) using the Logic-e (GE healthcare). Exemplary echocardiographic tracing of sPAP measurements are shown in the Supplementary Figures S1(a)–(d). Human endothelin-1 was quantified with QuantiGlo Elisa (R&D systems Inc, Minneapolis, USA) following the manufacturer's instructions.

### 2.4. Statistics

Statistical analyses were performed using GraphPad 7 (LaJolla, California, USA). The Shapiro–Wilk test was used to test for normal distribution. Because data passed this test, parametric tests were used throughout the study (*t*-test, Pearson correlation). A *p* < 0.05 was considered significant. As this was a pilot study, we did not perform correction for multiple testing and also reported tendencies (*p* < 0.1).

## 3. Results

The median age of subjects in the study was 34 years in both groups (Table 1). Further baseline characteristics including body mass index (BMI), physical activity, and preexisting medical conditions were not significantly different between the groups. The median duration of exposure to CIHH was 3.7 years (minimum 2.5 years and maximum 20 years; Table 1).

All subjects had a normal lung function and diffusion capacity with no significant differences between groups (Table 2). Baseline laboratory at low altitude showed a tendency toward higher Hb in CIHH subjects (*p*=0.05). Pro-BNP levels were normal in all subjects and did not show significant difference between the groups.

At low altitude, resting echocardiography showed normal right and left cardiac dimensions and function and absence of cardiac disease in all subjects (Table 3). Of note, right heart function as measured by tricuspid annular plane systolic excursion (TAPSE) was similar in both groups. All subjects had either normal sPAP at rest or it was not possible to measure due to lack of tricuspid regurgitation (Table 3). sPAP at rest was not significantly different between CIHH subjects and controls. Low-altitude blood gas analysis at rest revealed a tendency toward increased hyperventilation with lower pCO_2_ and decreased base excess (BE) in CIHH subjects (Table 3). During exercise at 150 W, CIHH subjects had a significantly lower sPAP and higher SpO_2_ than controls, whereas TAPSE did not differ (Table 3).

**Figure 2:**
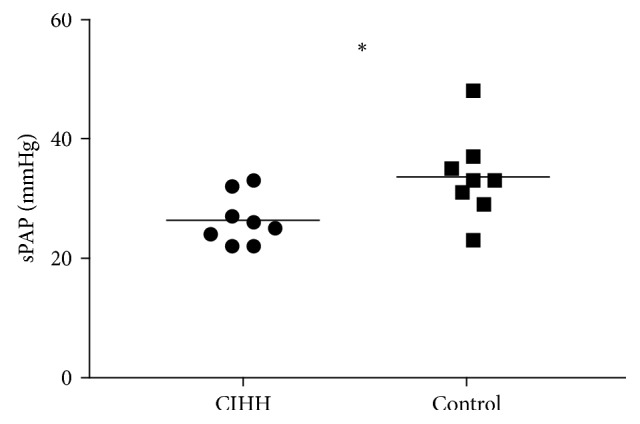
sPAP under exertion (75 W) at high altitude. sPAP was significantly higher in CIHH than in controls. CIHH: chronic intermittent hypobaric hypoxia. ^∗^By *t*-test.

At high altitude, there was a significantly lower pCO_2_ at rest in CIHH subjects compared to controls, and BE tended to be lower (Table 4). Further, pO_2_ at rest at high altitude tended to be higher with longer duration of exposure to CIHH (Supplementary Figure S2). Upon treadmill exercise at high altitude with 75 W, sPAP was significantly lower in CIHH subjects while SpO_2_ was significantly higher than in controls (Figure 2 and Table 4). Again TAPSE was not significantly different between CIHH and control subjects. pCO_2_ tended to be lower at exercise at high altitude (Supplementary Table 1); however, exercise BGA was not obtained at the same power level in all subjects.

**Figure 3:**
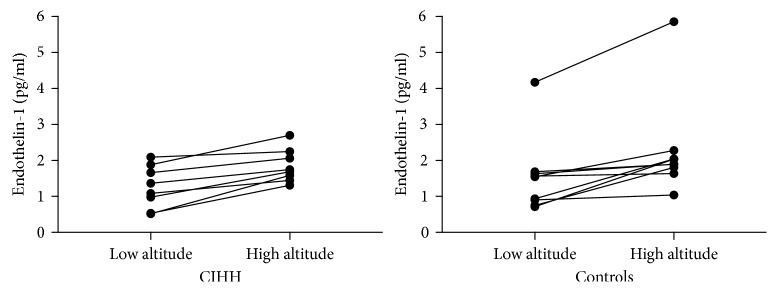
Endothelin-1 at lowland and high altitude. Endothelin-1 serum levels increased at high altitude compared to lowland in CIHH as well as in controls (*p* < 0.01 in both groups, by *t*-test, paired). There was no significant difference between CIHH and controls. Measurements at lowland and altitude from each individual are connected by a line.

As endothelin-1 has been shown to be of importance for hypoxic pulmonary vasoconstriction and chronic hypoxic PH [19], it was quantified in serum in low and high altitude. Here, endothelin-1 was increased at high altitude compared to low altitude in CIHH as well as in control subjects (Figure 3, *p* < 0.01). However, there were no significant differences between the groups. Similarly, pro-BNP was increased at high altitude compared to low altitude in the total study population (*p*=0.02), but there were no significant differences between the groups (Tables 2 and 4).

**Figure 4:**
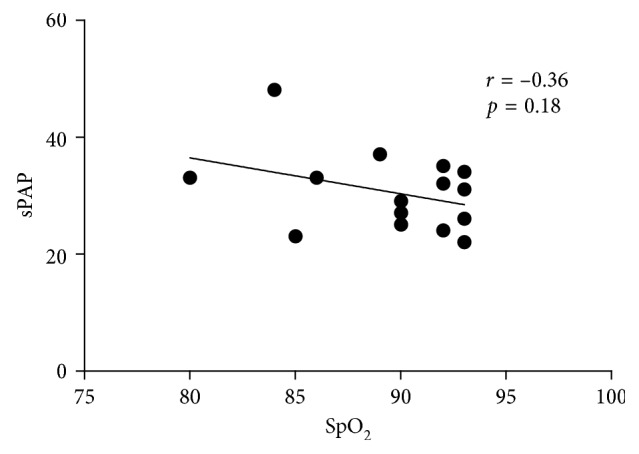
Correlation of sPAP and SpO_2_ under exertion at high altitude. Each data point corresponds to one subject (CIHH or control). Correlation was not statistically significant.

Since sPAP was lower and SpO_2_ higher in CIHH than in controls, we further investigated a possible correlation between sPAP and SpO_2_ during exercise at high altitude and found a negative correlation that was not statistically significant (Figure 4).

## 4. Discussion

To our knowledge, this is the first study investigating the influence of daily CIHH on pulmonary arterial pressure. In contrast to chronic exposure to hypoxia, daily CIHH at an altitude of 2650 m did not lead to increases in sPAP compared to constant normobaric normoxia in lowlanders. Single breath diffusion capacity as well as Hb corrected Krogh factor were normal and similar to controls. Thus, there were no signs to suggest significant vascular remodeling in the CIHH subjects studied. Moreover, no signs of remodeling or altered function of the right heart were found.

While studies of chronic exposure have consistently shown chronic increases in PAP in high-altitude dwellers [2, 11, 20], results obtained for CIHH are less clear [14–16, 21, 22]. Antezana et al. prospectively investigated Chilean miners exposed to weekly CIHH at 4500 m asl for 3 years and found no signs of PH apart from increased PAP during hypoxia [21]. In this setting, PAP was lower in CIHH subjects than in permanent residents [14]. In a study of monthly CIHH at 3700 m in Kyrgyzstan over two years, similar results were obtained [21]. Yet, in an observational study investigating subjects exposed to weekly CIHH at 3550 m for more than a decade, Brito et al. found echocardiographic signs of elevated PAP in 6 out of 50 subjects [16]. From these studies, it can be concluded that pulmonary vascular remodeling may occur in CIHH as well but possibly only after very long exposure duration for more than a decade. In contrast, in the present study we did not find signs of chronic increases in PAP or remodeling in any of the subjects. This may be due to a shorter exposure interval of daily versus weekly exposure but also a lower height of 2650 m versus 3700 m. Chronic hyperventilation in adapted subjects at 2650 m might still be sufficient to maintain pO_2_ at a level that prevents pronounced hypoxic PAP increase, whereas this compensation might not be sufficient at a very high altitude resulting in long-term remodeling. Moreover, we included only two subjects with very long exposure duration over 10 years, and therefore, it cannot be excluded that in a larger population subjects with signs of PH may be found. Interestingly, pO_2_ at rest at high altitude tended to be higher after longer exposure to CIHH, whereas dampening of HVR is observed in native Andean populations [23].

Of note, in the present study sPAP under exertion at low and high altitude was even lower in CIHH subjects compared to controls. One possible explanation for this is adaptation. Firstly, we observed moderately higher hemoglobin, possibly reflecting the adaptation to the chronic intermittent high-altitude exposure and confirming a relevant but moderate exposure to hypoxia. The Hb values we found were lower than what would be expected from chronic exposure to a similar height, in line with previous studies of CIHH [14, 16]. Secondly, pCO_2_ was lower both at low and at high altitude while BE was lower and pH was unchanged as a sign of chronic hyperventilation as means of ventilatory adaptation. More pronounced hyperventilation increases pO_2_, possibly resulting in reduced hypoxic vasoconstriction and therefore explaining the slightly lower sPAP that was observed. Even though SpO_2_ under exertion in both, high altitude and low altitude, was significantly higher in CIHH subjects compared to controls, a direct correlation between sPAP and SpO_2_ could not be demonstrated. The reason for this could be the small number of subjects studied, but other confounding factors such as individual differences based on genetic predisposition or differences in vasoactive mediators or differences in cardiac output are also possible. Moreover, results of BGA at exercise are not conclusive, possibly because they were not measured at the same power level in all subjects (Supplementary Table S1).

Cardiac output was not measured in this study, but right heart function during exercise was assessed by TAPSE measurement. Here, we found that TAPSE in CIHH was increased as compared to resting measurement and not significantly different from controls at low as well as high altitude. We did not find any signs of decreased right heart function compared to controls. We further studied some of the vasoactive mediators that have been involved in the control of hypoxic pulmonary vasoconstriction and have also been linked long-term changes such as remodeling of the pulmonary vasculature and right heart. Brain natriuretic peptide (BNP) has diuretic, natriuretic, and hypotensive effects with cytoprotective and antiproliferative properties. It is clinically used as a marker of ventricular load, especially right ventricular load in pulmonary hypertension. Cardiac BNP release is increased under hypobaric hypoxia [24, 25], and disproportionately high increase of pro-BNP has been shown in HA pulmonary edema [26]. Endothelin-1 is a potent vasoconstrictor but also promotes vascular remodeling with migration and proliferation of vascular cells [24]. It increases in response to acute hypoxia [27] and hypoxia-induced PAP increase may be blunted by endothelin receptor blockade [28]. In our study, pro-BNP and endothelin-1 were increased at high altitude compared to low altitude but there were no significant differences between CIHH and control subjects. This supports our finding that there is no sign of chronic PH in CIHH. Further, there seems to be no adaptation to the HA exposure on this level.

Surprisingly, in our study hyperventilation was observed in CIHH subjects even at low altitude. Minute ventilation is increased in response to hypoxia by a rise in breathing frequency and tidal volume. This hypoxic ventilator response is triggered by the chemoreceptors in the bifurcation of the carotid arteries. In a study using 1 h of hypoxia per day for one week, it was found that HVR increased via augmented hypoxic sensitivity of chemoreceptors [29]. This enhanced HVR persisted after return to sea level for one week. In another study by Ricart et al., subjects were exposed to an altitude of 5000 m for 2 h per day for 2 weeks and subsequently exercised during hypoxia [30]. Here, it was found that ventilation and arterial oxygenation during exercise were higher than before the intermittent hypoxia and this is in line with our results. However, in that study resting ventilation was not altered, whereas we found hyperventilation also at rest even in lowland. Our low-altitude measurements were performed after all subjects had spent the night at normoxia as usual indicating hyperventilation is sustained for at least this time period without hypoxic stimulus.

This study should be interpreted in view of its limitations. It is an observational study of subjects exposed to daily CIHH for several years using matched controls for comparison. Therefore, subjects working at high altitude for years might be selected for those who adapt well and not correspond to the general population.

Further, in this pilot study we investigated a small number of subjects, and larger studies will be necessary to confirm the results obtained. Moreover, we used noninvasive assessment of PAP as well as heart structure and function. Invasive measurement of pulmonary hemodynamics is the gold standard providing a more exact and complete picture with measurement of CO and PVR in addition to PAP. However, reliability of echocardiographic sPAP measurement is high [31] and negative predictive value to exclude PH is high when parameters of RV structure and function and BNP are also considered [32]. Additionally, we used high-altitude exposure and exercise as challenges, and thus we are confident these results proof valid. Moreover, our techniques are comparable to other studies on this matter which also used echocardiographic assessment. In sum, the reliability of the finding of normal sPAP and right heart function in CIHH is high, whereas it is lower for the small differences in sPAP found between CIHH and controls warranting further investigations using more precise methods. Finally, we have not directly measured minute ventilation but can only conclude from pCO_2_ levels and BE. Future studies should include more detailed and direct measurement of ventilation.

## 5. Conclusions and Outlook

Daily CIHH to an altitude of 2650 m leads to adaptation with moderately higher hemoglobin and chronic hyperventilation with higher O_2_ saturation during exercise but no disproportionate increase in sPAP even during exercise. Meanwhile, there were no signs of chronic increase in PAP or altered RV structure and function. Larger studies are necessary to confirm results and obtain more insights into degree and mechanism of chronic hyperventilation that seems to persist even at lowland.

## Figures and Tables

**Table 1:** Baseline characteristics.

Matched characteristics	CIHH	Controls	*p* value^∗^
Total subject number, *n*	8	8	
Female, *n*	4	4	
Age, years median (min; max)	34 (29; 50)	34.5 (28; 51)	
BMI, kg/m^2^ (±SD)	25.30 ± 3.78	23.90 ± 6.19	0.57
Smoking (current/ex/never)	2/2/4	0/1/7	0.21
Packyears			
Current	8; 16		
Ex-smoker	0.5; 2	12	
Endurance sport activity (regular/sometimes/rarely)	2/6/0	2/5/1	0.58
Treated hypertension, *n*	2	2	
Treated hypothyreosis, *n*	0	1	>0.99
Duration of CIHH, years		—	
(i) Median (min; max)	3.7 (2.5; 20)		
(ii) Individual values	2.5, 3, 3.2, 3.4, 4, 5, 10, 20		

CIHH: chronic intermittent hypobaric hypoxia; ^∗^by *t*-test or chi-square test as appropriate. Endurance physical activity was defined as cardiac workout of at least 30 min: regular >1/week; sometimes 1–3x/month; rarely <1/month.

**Table 2:** Lowland pulmonary function tests and laboratory parameters.

Pulmonary function test	CIHH^#^	Control^#^	*p* value^∗^
FEV1 pp	104.29 ± 5.85	110.56 ± 16.83	0.34
FVC pp	110.70 ± 12.99	111.01 ± 8.71	0.96
FEV1/FVC	78.30 ± 5.37	81.99 ± 5.39	0.19
MEF 75% pp	90.23 ± 19.50	107.57 ± 34.6	0.23
MEF 50% pp	82.6 ± 11.32	105.3 ± 40.65	0.17
MEF 25% pp	75.25 ± 14.47	88.48 ± 38.07	0.37
R tot (kPa^∗^s/L)	0.20 ± 0.10	0.20 ± 0.07	0.95
TLC pp	107.68 ± 12.00	92.87 ± 36.78	0.30
DLCO-SB pp	97.69 ± 14.59	90.18 ± 11.41	0.27
DLCO/VA pp	97.41 ± 16.94	88.77 ± 12.01	0.26
DLCOc/VA pp	93.16 ± 15.28	88.45 ± 9.50	0.47
*Baseline laboratory*			
Hb (mg/dl)	14.95 ± 0.95	13.81 ± 1.28	**0.05**
Ferritin (*µ*g/l)	127.25 ± 131.23	132.63 ± 119.47	0.93
Pro-BNP (pg/ml)	17.80 ± 13.48	39.39 ± 38.60	0.18
Endothelin-1 (pg/ml)	1.26 ± 0.59	1.54 ± 1.06	0.73

CIHH: chronic intermittent hypobaric hypoxia; FEV1: forced expiratory volume in 1 sec; pp: percent predicted; FVC: forced vital capacitiy; R: resistance; TLC: total lung capacity; DLCO: lung diffusion capacity for carbon monoxide; SB: single breath; VA: alveolar volume; c: corrected for Hb; Hb: hemoglobin; BNP: brain natriuretic peptide; MEF 75, 50, 25: maximal expiratory flow at 25/50/75% of forced VC; ^∗^by *t*-test; ^#^±SD.

**Table 3:** Lowland echocardiography and blood gas analysis at rest and during exercise (*p*=150  W).

	CIHH	Control	*p* value^∗^
*Echocardiography at rest*			
LVEDD (mm)	45.50 ± 3.42	43.75 ± 2.82	0.28
LV-EF (%)	67.63 ± 2.39	65.57 ± 2.37	0.11
RVEDD (mm)	35.25 ± 3.20	32.88 ± 2.36	0.11
sPAP, not measurable at rest (*n*)^#^	3	4	
sPAP (mmHg)^#^	20.20 ± 3.27	18.50 ± 2.08	0.39
TAPSE (mm)	23.88 ± 3.48	22.67 ± 2.58	0.48
*Vital signs rest lowland*			
HR (bpm)	78.28 ± 13.64	77.5 ± 11.64	0.91
Mean RR (mmHg)	94.95 ± 10.37	92.83 ± 8.31	0.69
*Blood gas analysis rest lowland*			
pO_2_ (mmHg)	86.41 ± 4.55	85.49 ± 5.91	0.73
pCO_2_ (mmHg)	33.63 ± 4.63	37.65 ± 3.17	**0.06**
pH	7.43 ± 0.03	7.43 ± 0.02	1.00
BE (mmol/l)	−0.90 ± 2.21	0.64 ± 1.08	**0.09**
SaO_2_ (%)	97.29 ± 0.43	97.00 ± 0.74	0.37
AaDO_2_	21.2 ± 5.5	17.1 ± 6.1	0.18
*Exertion 150* *W*			
HR (bpm)	134.42 ± 12.71	142.5 ± 21.35	0.41
Mean RR (mmHg)	120.58 ± 13.66	110.27 ± 19.14	0.26
sPAP (mmHg)	28.75 ± 3.99	35.33 ± 2.89	**0.02**
TAPSE (mm)	30.00 ± 2.83	31.17 ± 5.23	0.59
SpO_2_ (%)	99.33 ± 1.21	94.75 ± 2.60	**0.001**

CIHH: chronic intermittent hypobaric hypoxia; LVEDD: left ventricular end diastolic diameter; LV-EF: left ventricular ejection fraction; RVEDD: right ventricular end diastolic diameter; sPAP: systolic pulmonary arterial pressure; TAPSE: tricuspid annular plane systolic excursion; pO_2_: oxygen partial pressure; pCO_2_: carbon dioxide partial pressure; BE: base excess; SaO_2_: arterial oxygen saturation; SpO_2_: peripheral oxygen saturation;^∗^by *t*-test; ^#^due to lack of TI, sPAP was not measurable in all subjects at rest at lowland; mean sPAP is therefore calculated from the remaining subjects only. During exercise and at altitude, sPAP was measurable in all subjects.

**Table 4:** High-altitude echocardiography and blood gas analysis at rest and during exercise (*p*=75  W).

High altitude	CIHH	Control	*p* value^∗^
*Laboratory*			
Pro-BNP (pg/ml)	39.5 ± 27.2	43.0 ± 41.2	0.85
Endothelin-1 (pg/ml)	1.84 ± 0.45	2.27 ± 1.38	0.37
*Echocardiography at rest*			
sPAP, not measurable at rest (*n*)	0	0	
sPAP (mmHg)	19.6 ± 3.3	20.1 ± 4.1	0.82
TAPSE (mm)	21 ± 3.9	22.1 ± 4.5	0.80
*Vital signs at rest altitude*			
HR (bpm)	79.4 ± 21	88.9 ± 22.2	0.41
Mean RR (mmHg)	104.0 ± 9.4	98.4 ± 10.8	0.31
*Blood gas analysis at rest altitude*			
pO_2_ (mmHg)	70.1 ± 7.3	63.2 ± 7.6	0.10
pCO_2_ (mmHg)	28.5 ± 2.3	31.8 ± 2.4	**0.01**
pH	7.44 ± 0.01	7.44 ± 0.01	0.46
BE (mmol)	−3.64 ± 1.12	−2.30 ± 1.32	**0.05**
SaO_2_ (%)	93.1 ± 1.0	91.4 ± 2.5	0.10
AaDO_2_	8.1 ± 7.6	11.2 ± 6.8	0.43
*Echocardiography exertion 75* *W*			
sPAP (mmHg)	26.3 ± 4.1	33.6 ± 7.2	**0.04**
TAPSE (mm)	26.2 ± 2.8	25.6 ± 4.6	0.80
SpO_2_ (%)	91.83 ± 1.47	87.38 ± 4.41	**0.03**

CIHH: chronic intermittent hypobaric hypoxia; sPAP: systolic pulmonary arterial pressure; TAPSE: tricuspid annular plane systolic excursion; pO_2_: oxygen partial pressure; pCO_2_: carbon dioxide partial pressure; BE: base excess; SaO_2_: arterial oxygen saturation; SpO_2_: peripheral oxygen saturation; ^∗^by *t*-test.

## Data Availability

Source data are available via Open Science Framework (https://osf.io/p4nrx/).

## Supplementary Materials

Supplementary Materials Supplementary Figure S1: exemplary echocardiographic tracings of sPAP measurements. (a) Lowland at rest. (b) Lowland exercise. (c) High altitude at rest. (d) High-altitude exercise. Supplementary Figure S2: correlation between pO_2_ at high altitude at rest and years exposure to CIHH. Supplementary Table S1: BGA under exertion at lowland and high altitude.
