# Supplementary material for: Daily Chronic Intermittent Hypobaric Hypoxia Does Not Induce Chronic Increase in Pulmonary Arterial Pressure Assessed by Echocardiography
Source: Can Respir J. 2018 Apr 1;2018:9649716. doi: 10.1155/2018/9649716 (PMC5902055; doi:10.1155/2018/9649716)
Supplement: Supplementary Materials — Supplementary Figure S1: exemplary echocardiographic tracings of sPAP measurements. (a) Lowland at rest. (b) Lowland exercise. (c) High altitude at rest. (d) High-altitude exercise. Supplementary Figure S2: correlation between pO2 at high altitude at rest and years exposure to CIHH. Supplementary Table S1: BGA under exertion at lowland and high altitude. [file 9649716.f1.docx]

**Daily chronic intermittent hypobaric hypoxia does not induce chronic increase in pulmonary arterial pressure assessed by echocardiography**

Jeremias Götschke^1,2^*, Pontus Mertsch^1,2^*, Nikolaus Kneidinger^1,2^, Diego Kauffmann-Guerrero^1,2^, Jürgen Behr^1,2,3^, Rudolf Maria Huber^1,2^, Frank Reichenberger^2,3^, Katrin Milger^1,2^

*contributed equally

**c**

**Supplementary Figure S2 Correlation between pO2 at high altitude at rest and years of exposure to CIHH.**

**
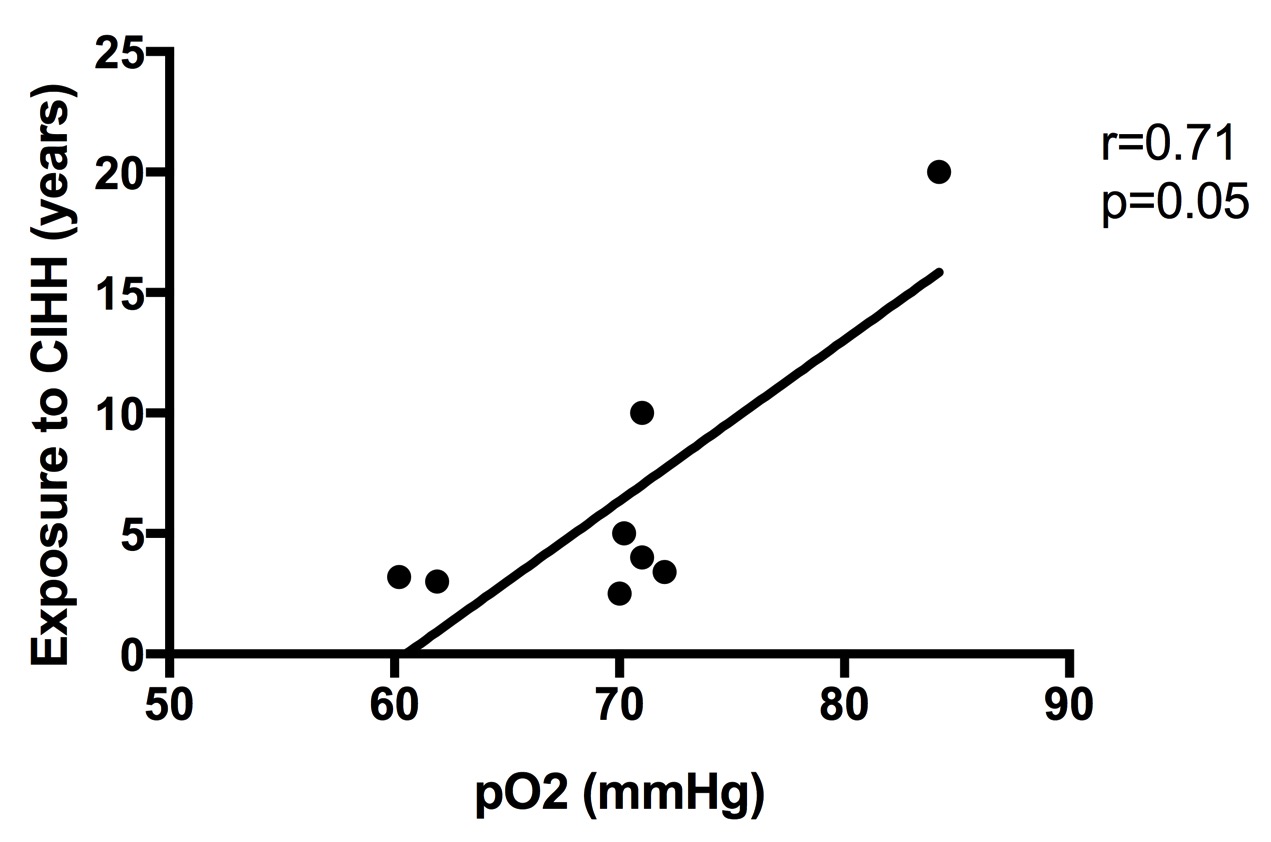
**

**Supplementary Table S1 BGA under exertion at lowland and high-altitude**

| BGA exertion | CIHH | Control | P-Value * |
| --- | --- | --- | --- |
| Lowland |  |  |  |
| pO_2_ – mmHg | 89.7 ± 6.0 | 86.8 ± 6.17 | 0.35 |
| pCO_2_ – mmHg | 34.1 ± 3.5 | 37.5 ± 4.09 | 0.09 |
| pH | 7.34 ± 0.02 | 7.32 ± 0.03 | 0.13 |
| AaDO2– mmHg | 9.0 ± 5.7 | 8.3 ± 6.2 | 0.82 |
| High-altitude |  |  |  |
| pO_2_– mmHg | 64.2 ± 1.1 | 61.0 ± 4.2 | 0.06 |
| pCO_2_– mmHg | 28.2 ± 3.7 | 29.9 ± 2.8 | 0.32 |
| pH | 7.34 ± 0.02 | 7.34 ± 0.04 | 0.81 |
| AaDO2– mmHg | 14.7 ± 5.0 | 16.0 ± 4.9 | 0.63 |

AaDO2= Alveolar-arterial oxygen difference. It was planned to obtain BGA under exertion at 200W and 100W for lowland and high-altitude respectively. However, as some subjects did not achieve this power, it was measured at 150W and 75W in these subjects (3 subjects in the CIHH and 5 subjects in the control group). As all other measurements including sPAP, TAPSE, SpO2 were performed at each power level, all other analyses were performed at 150W and 75W in order to facilitate comparison between groups.
